# Thrombotic Complications in Women Undergoing Assisted Reproductive Technologies: Pathophysiology, Prevention, and Clinical Management—A Narrative Review

**DOI:** 10.3390/hematolrep18050077

**Published:** 2026-10-16

**Authors:** Carmine Siniscalchi, Augusto Delle Femine, Ciro Di Gennaro, Manuela Basaglia, Mariano Galdiero, Antonio Lobasso, Egidio Imbalzano, Antonella Tufano, Pierpaolo Di Micco

**Affiliations:** 1 Internal Medicine Ward, “Santa Maria delle Grazie” Hospital, 80078 Pozzuoli, Naples, Italy; 2 Internal Medicine Ward, Parma University Hospital, 43125 Parma, Italy; augusto.dellefemine@aslnapoli2nord.it (A.D.F.); ciro.digennaro@aslnapoli2nord.it (C.D.G.); mbasaglia80@gmail.com (M.B.); mariano.galdiero@aslnapoli2nord.it (M.G.); antonio.lobasso@aslnapoli2nord.it (A.L.); pdimicco@libero.it (P.D.M.); 3 Department of Clinical and Experimental Medicine, University of Messina, 98122 Messina, Italy; egidio.imbalzano@unime.it; 4 Department of Clinical Medicine and Surgery, Federico II University of Naples, 80121 Naples, Italy; atufano@unina.it

**Keywords:** assisted reproductive technologies, in vitro fertilization, ovarian hyperstimulation syndrome, thrombophilia, venous thromboembolism, thromboprophylaxis

## Abstract

Thrombotic complications represent uncommon but potentially life-threatening adverse events associated with assisted reproductive technologies (ARTs), particularly in the setting of controlled ovarian stimulation and ovarian hyperstimulation syndrome (OHSS). The relationship between thrombophilia, infertility, ovarian hyperstimulation syndrome (OHSS), and venous thromboembolism (VTE) remains complex and only partially understood. While antiphospholipid syndrome is a well-established acquired thrombophilic disorder associated with recurrent pregnancy loss, the association between common inherited thrombophilias and recurrent pregnancy loss is less consistent, and their contribution to primary infertility and implantation failure remains uncertain. Conversely, severe OHSS is consistently recognized as the principal treatment-related determinant of thrombotic events during IVF because of profound endothelial activation, hemoconcentration, and hypercoagulability. This narrative review summarizes the current evidence regarding the epidemiology, pathophysiology, and clinical presentation of thrombotic complications associated with assisted reproductive technologies, including deep vein thrombosis, pulmonary embolism, cerebral venous thrombosis, upper-extremity thrombosis, splanchnic vein thrombosis, and arterial thrombotic events. Current recommendations for thromboprophylaxis, diagnostic strategies, and therapeutic management are critically discussed, with particular attention to individualized risk assessment and the role of low-molecular-weight heparin in high-risk patients. Although routine thromboprophylaxis is not recommended for all women undergoing in vitro fertilization (IVF), careful evaluation of patient-specific thrombotic risk factors remains essential. Future prospective studies are needed to improve risk prediction, optimize preventive strategies, and establish evidence-based recommendations for women undergoing assisted reproduction.

## 1. Introduction

Over the past three decades, assisted reproductive technologies (ARTs), particularly in vitro fertilization (IVF), have become an integral component of infertility management worldwide. Delayed childbearing, declining fertility rates, improvements in laboratory techniques, and broader indications for fertility preservation have all contributed to a steady increase in the number of ART cycles performed annually. According to the European IVF-monitoring Consortium (EIM) of the European Society of Human Reproduction and Embryology (ESHRE), more than one million treatment cycles are currently performed each year in Europe alone, and this number continues to rise, making even uncommon complications increasingly relevant in everyday clinical practice [1].

Female infertility is a multifactorial condition resulting from gynecological, endocrine, immunological, genetic, and systemic disorders. Antiphospholipid syndrome (APS) is a well-established acquired thrombophilic disorder associated with recurrent pregnancy loss and other adverse obstetric outcomes. In contrast, the association between common inherited thrombophilias and recurrent pregnancy loss is considerably less consistent, and their role in primary infertility or implantation failure remains uncertain. Consequently, routine inherited thrombophilia screening is not recommended in all women undergoing IVF and should be guided by the individual clinical and thrombotic history. Antiphospholipid antibody testing should instead be considered according to established criteria for suspected APS, particularly in women with appropriate obstetric or thrombotic manifestations [2,3,4,5].

A clear distinction should also be maintained between inherited thrombophilia and APS, which is an acquired autoimmune thrombophilic disorder with different therapeutic implications. In women with obstetric APS, current recommendations generally support low-dose aspirin combined with prophylactic-dose heparin or low-molecular-weight heparin (LMWH), whereas women with a history of thrombotic APS generally require low-dose aspirin combined with therapeutic-dose heparin or LMWH during pregnancy. Thus, management should be guided by the clinical APS phenotype and previous thrombotic history rather than by applying a uniform anticoagulant regimen to all patients with thrombophilia [6].

More broadly, the prothrombotic phenotype of APS reflects the interaction between autoantibodies, endothelial and platelet activation, complement-mediated inflammation, and immune–coagulation crosstalk, further supporting its biological distinction from conventional inherited thrombophilia.

Conversely, common MTHFR polymorphisms should not currently be considered established thrombophilic determinants of ART-related thrombosis or reproductive failure. Although interactions among folate metabolism, autoimmune disorders, and reproductive outcomes have been investigated, available evidence does not support routine MTHFR genotyping for thrombophilia assessment in women undergoing ARTs.

The relationship between thrombophilia, assisted reproduction, and antithrombotic therapy therefore remains complex. On one hand, thrombophilic disorders may contribute to adverse reproductive outcomes in selected women; on the other hand, ovarian stimulation itself induces profound hormonal and hemostatic changes that transiently increase thrombotic risk. The development of ovarian hyperstimulation syndrome (OHSS) further amplifies this prothrombotic state through endothelial activation, hemoconcentration, increased vascular permeability, and activation of the coagulation cascade, making OHSS the principal treatment-related determinant of thrombosis during ARTs [3,4,5,6,7].

Although the absolute incidence of thrombotic complications remains low, women undergoing IVF experience a significantly higher risk of venous thromboembolism (VTE) than women with spontaneous conception, particularly during the first trimester of pregnancy and in the presence of severe OHSS [2]. Most thrombotic events are venous, including deep vein thrombosis (DVT) and pulmonary embolism (PE); however, thrombosis involving atypical sites such as the upper-extremity veins, cerebral venous sinuses, and splanchnic circulation has been reported more frequently than in spontaneous pregnancies [5].

Another evolving area concerns heterologous IVF, including oocyte and embryo donation. Women undergoing these procedures are frequently older and present with a greater burden of cardiovascular risk factors than those receiving homologous IVF. Whether the reproductive technique itself contributes to thrombotic risk or whether the increased incidence of thrombotic events primarily reflects patient characteristics remains uncertain and deserves further investigation.

The increasing number of ART procedures performed worldwide highlights the importance of recognizing thrombotic complications promptly, identifying women who may benefit from thromboprophylaxis, and optimizing multidisciplinary management. The major clinical factors associated with thrombotic risk during ARTs are summarized in Table 1.

The aim of this narrative review is to provide an updated overview of the relationship between thrombophilia, assisted reproductive technologies, and thrombotic complications, with particular emphasis on the mechanisms underlying hypercoagulability, the clinical spectrum of venous and arterial thrombosis, current recommendations for thromboprophylaxis, and future perspectives for individualized thrombotic risk stratification.

## 2. Materials and Methods

This narrative review was based on a literature search conducted in PubMed/MEDLINE and Scopus, including articles published from database inception to September 2026. The search strategy combined terms related to assisted reproduction and thrombotic complications using the following search string: (“assisted reproductive technology” OR “in vitro fertilization” OR “ovarian stimulation” OR “ovarian hyperstimulation syndrome”) AND (“venous thromboembolism” OR “deep vein thrombosis” OR “pulmonary embolism” OR “cerebral venous thrombosis” OR “splanchnic vein thrombosis” OR “arterial thrombosis” OR “thrombophilia” OR “antiphospholipid syndrome” OR “thromboprophylaxis”). Additional relevant publications were identified through manual screening of the reference lists of selected articles.

Priority was given to clinical guidelines, systematic reviews, meta-analyses, randomized controlled trials, and large observational studies. Case reports and case series were considered when addressing rare or unusual thrombotic manifestations for which higher-level evidence was unavailable. Evidence specifically addressing ARTs and OHSS was prioritized; however, because ART-specific evidence remains limited for several clinical scenarios, pregnancy-associated and general VTE literature was also considered when directly relevant.

The literature search and selection of relevant publications were independently performed by C.S. and P.D.M., with disagreements resolved by discussion. Given the narrative design of the review, no formal systematic risk-of-bias assessment, quantitative evidence synthesis, or assessment of the certainty of evidence using the Grading of Recommendations Assessment, Development and Evaluation (GRADE) framework was performed.

## 3. Hypercoagulable State and Thromboprophylaxis in Ovarian Hyperstimulation Syndrome

The thrombotic relevance of ovarian hyperstimulation syndrome (OHSS) derives from the profound hemodynamic, endothelial, and coagulation changes occurring in its severe forms. Although its incidence has progressively declined owing to contemporary ovarian stimulation protocols, severe OHSS remains an important treatment-related determinant of thrombotic complications during ARTs [3,4]. The principal clinical and patient-related risk factors associated with ART-related thrombosis are summarized in Table 1.

Importantly, contemporary prevention of ART-associated thrombosis begins with strategies aimed at preventing or attenuating OHSS itself. In women predicted to have a high ovarian response, gonadotropin-releasing hormone (GnRH)-antagonist protocols permit the use of a GnRH-agonist trigger instead of conventional human chorionic gonadotropin (hCG) triggering, thereby reducing prolonged luteotropic stimulation and the risk of clinically significant OHSS. Freeze-all strategies, in which all embryos are cryopreserved and transfer is deferred to a subsequent cycle, further reduce the risk of late OHSS by avoiding endogenous hCG exposure associated with an immediate pregnancy. Randomized evidence in high responders has supported the safety rationale of freeze-all strategies compared with fresh transfer protocols requiring additional low-dose hCG for luteal support [8]. More broadly, a recent umbrella review identified individualized stimulation, GnRH-antagonist protocols, GnRH-agonist triggering, freeze-all strategies, and selected pharmacological interventions as central components of contemporary OHSS prevention [9]. Dopamine agonists, particularly cabergoline, may also reduce the incidence of moderate-to-severe OHSS, probably through modulation of VEGF-mediated vascular permeability [10]. Because severe OHSS is the major treatment-related determinant of thrombosis during ARTs, these reproductive strategies should also be regarded as indirect thrombotic-risk reduction measures.

The pathophysiology of OHSS is complex and multifactorial. Following controlled ovarian stimulation, administration of human chorionic gonadotropin (hCG) induces ovarian secretion of vascular endothelial growth factor (VEGF), a key mediator responsible for the increased vascular permeability that characterizes the syndrome. The resulting extravasation of protein-rich fluid into the third space leads to ascites, pleural effusion, intravascular volume depletion, hemoconcentration, reduced venous flow, and increased blood viscosity, thereby fulfilling several components of Virchow’s triad and creating a profoundly prothrombotic environment [11].

In addition to hemoconcentration, OHSS is characterized by significant activation of the coagulation system. Elevated plasma concentrations of fibrinogen, factor VIII, von Willebrand factor, thrombin-antithrombin complexes, prothrombin fragment 1 + 2, and D-dimer have been consistently reported, together with impaired fibrinolysis and increased platelet activation [12,13,14]. These alterations are further amplified by endothelial dysfunction and the release of pro-inflammatory cytokines, supporting the concept that OHSS is not merely a hypercoagulable condition but also a thrombo-inflammatory syndrome.

At the cellular level, the prothrombotic phenotype associated with OHSS results from a dynamic interaction among endothelial cells, platelets, leukocytes, and the coagulation system. VEGF-mediated endothelial activation increases vascular permeability and promotes the expression of adhesion molecules and tissue factor, thereby facilitating leukocyte recruitment and thrombin generation. Activated platelets adhere to dysfunctional endothelium and interact with circulating leukocytes, further amplifying coagulation and inflammatory signaling. Neutrophil activation may additionally promote the release of neutrophil extracellular traps (NETs), which provide a negatively charged scaffold for platelet adhesion, coagulation-factor activation, and fibrin deposition. Concurrent hemoconcentration increases the local concentration of circulating blood cells and coagulation proteins, while impaired fibrinolytic activity favors thrombus persistence. These mechanisms are particularly relevant to venous thrombosis, in which coagulation activation, fibrin formation, reduced venous flow, and blood stasis predominate. By contrast, arterial thrombosis is more strongly driven by endothelial injury or activation, platelet adhesion and aggregation, vasoconstriction, and high-shear thrombus formation. Thus, although venous and arterial events share a common background of endothelial dysfunction and systemic thrombo-inflammation during OHSS, the relative contribution of coagulation, stasis, and platelet activation differs between the two vascular compartments.

Women who develop severe OHSS have a markedly higher incidence of thrombotic events than those undergoing uncomplicated IVF cycles. Venous thrombosis accounts for the vast majority of complications and frequently involves unusual anatomical sites, including the upper-extremity veins, internal jugular vein, cerebral venous sinuses, and splanchnic circulation, whereas arterial thrombosis remains uncommon but potentially catastrophic [3,4]. Most thrombotic events occur during the first trimester of pregnancy, when the procoagulant effects of OHSS overlap with the physiological hypercoagulability of early gestation.

Current international guidelines recommend an individualized approach to thromboprophylaxis rather than routine anticoagulation for all women undergoing ARTs. Low-molecular-weight heparin (LMWH) remains the preferred pharmacological option in women with severe OHSS because of its favorable efficacy, predictable pharmacokinetics, and well-established maternal and fetal safety profile [15]. Additional indications for LMWH include a previous episode of VTE, high-risk inherited thrombophilia, antiphospholipid syndrome, severe obesity, prolonged immobilization, or the coexistence of multiple acquired thrombotic risk factors.

The role of low-dose aspirin remains less clearly defined. Although aspirin has been investigated as a strategy to reduce the incidence of OHSS and potentially improve implantation rates, current evidence does not support its routine use for the prevention of thrombotic complications during IVF [16]. Consequently, careful patient selection, individualized thrombotic risk assessment, and early recognition of OHSS continue to represent the cornerstone of prevention.

## 4. Thrombophilia, Antithrombotic Therapy, and Thromboprophylaxis in Female Infertility

Routine inherited thrombophilia screening before in vitro fertilization (IVF) is not recommended in unselected infertile women. Testing for inherited thrombophilia should be reserved for selected women in whom the result is expected to influence clinical management, particularly those with a personal history of VTE or, in selected circumstances, a strong family history of thrombosis. Recurrent pregnancy loss alone should not be considered a general indication for inherited thrombophilia screening. In contrast, testing for antiphospholipid antibodies should be considered separately in women with clinical features suggestive of APS, including recurrent pregnancy loss meeting established obstetric criteria or a history of thrombosis [17,18,19]. Available clinical evidence remains inconsistent, and most observational studies have failed to demonstrate a clear causal relationship between inherited thrombophilia and unsuccessful IVF cycles [18,20].

Low-molecular-weight heparin (LMWH) has been the most extensively investigated pharmacological intervention in women undergoing IVF. Besides its anticoagulant properties, LMWH has been proposed to exert several biological effects potentially favorable for embryo implantation, including modulation of trophoblast invasion, inhibition of complement activation, reduction in inflammatory cytokine production, and interactions with adhesion molecules at the maternal–fetal interface [21]. Nevertheless, randomized controlled trials and subsequent meta-analyses have produced conflicting results. At present, current evidence does not support the routine administration of LMWH in unselected women undergoing IVF solely to improve implantation, clinical pregnancy, or live birth rates [17,21].

Low-dose aspirin has also been investigated because of its antiplatelet activity and its potential to improve uteroplacental perfusion. Although early observational studies suggested possible benefits in ovarian response and implantation, more recent systematic reviews have failed to demonstrate consistent improvements in reproductive outcomes [22]. Consequently, aspirin should not be routinely prescribed during ARTs in women without specific cardiovascular or obstetric indications [22].

A complementary strategy in women at particularly high risk of excessive ovarian response is to minimize or avoid gonadotropin exposure. In vitro maturation (IVM), in which immature oocytes are retrieved with limited or no ovarian stimulation and subsequently matured in vitro, has therefore been investigated particularly in women with polycystic ovary syndrome (PCOS), a population at increased risk of OHSS. A randomized controlled trial comparing unstimulated IVM with conventional hyperstimulated IVF in women with PCOS demonstrated that avoidance of gonadotropin stimulation substantially reduces the biological conditions responsible for OHSS, although reproductive effectiveness must also be considered when selecting this strategy [23]. The updated 2025 Cochrane review further supports IVM as a potentially relevant alternative in selected women at high risk of OHSS, while emphasizing persisting uncertainty regarding comparative reproductive outcomes and the need for individualized treatment selection [24]. From a thrombosis perspective, the potential relevance of IVM is therefore primarily preventive and indirect: avoidance of marked ovarian hyperstimulation may prevent the endothelial dysfunction, hemoconcentration, and coagulation activation that constitute the major treatment-related pathway to ART-associated VTE.

Conversely, pharmacological thromboprophylaxis should be considered in women with clinically relevant thrombotic risk factors, according to the specific clinical scenario and applicable guideline recommendations. Previous VTE, high-risk inherited thrombophilia, antiphospholipid syndrome, severe OHSS, prolonged immobilization, severe obesity, and the coexistence of multiple acquired risk factors may increase thrombotic risk during ovarian stimulation and early pregnancy. However, the indication for LMWH, its timing, and dose intensity should be individualized according to the nature and magnitude of the underlying risk factors [17,25]. When pharmacological thromboprophylaxis is indicated, the optimal LMWH dose should also be considered. The randomized Highlow trial compared intermediate-dose with low-dose LMWH in pregnant and postpartum women with a previous history of VTE and did not demonstrate a clear reduction in recurrent VTE with routine intermediate-dose prophylaxis [26]. Although this trial was not specifically conducted in women undergoing ARTs, its findings are clinically relevant once pregnancy has been established and argue against routine escalation to intermediate-dose LMWH solely on the basis of a previous VTE. Dose intensity should therefore be individualized according to recurrence risk, body weight, additional thrombotic risk factors, and bleeding risk.

Patients presenting with concomitant superficial vein thrombosis and deep vein thrombosis exhibit distinct clinical characteristics and outcomes compared with isolated DVT, reinforcing the importance of individualized thrombotic risk assessment rather than uniform therapeutic strategies [27,28,29,30].

## 5. Pharmacological Treatment in IVF and Deep Vein Thrombosis of the Lower Limb

Lower-extremity deep vein thrombosis (DVT) represents an important manifestation of venous thromboembolism associated with ARTs, although thrombosis at unusual venous sites appears to be relatively overrepresented among reported ART/OHSS-associated events [31,32].

Venous thromboembolism represents the predominant thrombotic complication associated with assisted reproductive technologies (ARTs), with lower-extremity deep vein thrombosis (DVT) and pulmonary embolism (PE) accounting for a substantial proportion of events, together with the characteristic occurrence of thrombosis at unusual venous sites. A contemporary systematic review and meta-analysis confirmed that ARTs are associated with an increased risk of VTE, with risk being particularly concentrated in pregnancies complicated by ovarian hyperstimulation syndrome and during the first trimester [15]. This temporal pattern reflects the convergence of ovarian stimulation-induced hypercoagulability, hemoconcentration and endothelial activation with the physiological prothrombotic adaptations of early pregnancy.

The thrombotic risk also appears to differ according to embryo-transfer strategy. In a nationwide cohort study, pregnancies achieved after fresh embryo transfer were associated with a markedly increased incidence of both VTE and PE during the first trimester compared with natural conception, whereas no corresponding increase in first-trimester VTE was observed after frozen-thawed embryo transfer [31]. These findings are biologically consistent with the greater hormonal exposure and ovarian stimulation associated with fresh cycles and further support strategies that minimize OHSS in high-risk women. Nevertheless, the choice between fresh and frozen embryo transfer should primarily follow reproductive indications and individualized assessment rather than thrombotic risk alone.

Clinically, lower-extremity DVT during ARTs presents similarly to conventional DVT, with unilateral limb swelling, calf pain, warmth, erythema, and tenderness. However, symptoms may be subtle or masked by fluid retention associated with ovarian stimulation, potentially delaying diagnosis. Compression duplex ultrasonography remains the first-line diagnostic modality because of its excellent sensitivity for proximal DVT, widespread availability, and absence of ionizing radiation, making it particularly suitable for pregnant women [24]. In cases with persistent clinical suspicion despite negative ultrasonography, serial imaging or magnetic resonance venography may be considered.

The diagnostic role of D-dimer during ARTs deserves particular attention. Physiological activation of coagulation during controlled ovarian stimulation and pregnancy progressively increases circulating D-dimer concentrations, substantially reducing the specificity of conventional diagnostic thresholds. Consequently, an elevated D-dimer level is not diagnostic of VTE in this clinical setting and should be interpreted within an appropriate diagnostic algorithm integrating clinical probability and, when indicated, imaging findings [33].

Therapeutic anticoagulation should be initiated promptly once DVT has been confirmed. Low-molecular-weight heparin (LMWH) remains the treatment of choice because of its favorable pharmacokinetic profile, predictable anticoagulant effect, and inability to cross the placenta.

Overall, prompt diagnosis, early initiation of LMWH, and individualized evaluation of recurrence risk remain the cornerstones of lower-extremity DVT management during assisted reproduction. The principal thrombotic manifestations associated with ARTs and their recommended diagnostic and therapeutic approaches are summarized in Table 2.

## 6. Pharmacological Treatment in IVF and Pulmonary Embolism

Pulmonary embolism associated with ARTs may occur during or shortly after ovarian stimulation, particularly in the presence of OHSS, or after pregnancy has been established. This distinction is clinically relevant because diagnostic and therapeutic considerations related to pregnancy do not necessarily apply to thrombotic events occurring before conception.

The diagnosis of PE during pregnancy remains challenging because dyspnea, tachycardia, and mild respiratory discomfort frequently overlap with physiological gestational adaptations. Pleuritic chest pain, unexplained hypoxemia, syncope, or sudden hemodynamic deterioration should immediately raise clinical suspicion. Diagnostic algorithms should combine careful clinical assessment with compression ultrasonography of the lower limbs, ventilation–perfusion scintigraphy, or computed tomography pulmonary angiography (CTPA), balancing diagnostic accuracy against maternal and fetal radiation exposure. Recent diagnostic pathways integrating clinical probability assessment and adapted D-dimer strategies have improved the evaluation of suspected PE during pregnancy; however, their specific performance in women undergoing ARTs requires further validation [34].

Low-molecular-weight heparin (LMWH) remains the first-line treatment because of its predictable pharmacokinetics, favorable safety profile, and inability to cross the placenta. For PE occurring during pregnancy, therapeutic anticoagulation should generally be maintained throughout pregnancy and continued for at least six weeks after delivery, with an overall minimum duration of three months. For ART-associated PE occurring before pregnancy, treatment duration should instead follow general VTE recommendations and be individualized according to the provoking factors and recurrence risk. Routine monitoring of anti-factor Xa activity is unnecessary in most patients but may be considered in women with severe obesity, renal impairment, or extreme body weight.

Systemic thrombolysis should be reserved for high-risk PE complicated by shock or persistent hypotension, whereas catheter-directed interventions or surgical embolectomy may be considered in selected cases when thrombolysis is contraindicated or ineffective [35,36].

## 7. Pharmacological Treatment in IVF and Upper-Extremity Deep Vein Thrombosis

The mechanisms underlying the preferential involvement of the upper venous circulation remain incompletely understood. Severe hemoconcentration, profound endothelial dysfunction, activation of coagulation pathways, and increased blood viscosity induced by OHSS are believed to interact with local hemodynamic factors, ultimately favoring thrombus formation in venous territories that are otherwise infrequently affected [3,34]. Pregnancy itself further amplifies this prothrombotic milieu through physiological increases in coagulation factors, reduced fibrinolytic activity, and venous stasis [3,34].

Clinical presentation is frequently nonspecific. Patients may complain of unilateral arm swelling, neck pain, supraclavicular edema, cyanosis, shoulder discomfort, or visible superficial collateral veins [3]. Internal jugular vein thrombosis may initially present with isolated cervical pain or swelling, delaying diagnosis when clinical suspicion is low. Because these symptoms may easily be attributed to benign musculoskeletal conditions or fluid retention associated with ovarian stimulation, clinicians should maintain a high index of suspicion in women undergoing ARTs who develop persistent cervical or upper-limb symptoms [3].

Compression duplex ultrasonography remains the first-line diagnostic imaging modality because of its excellent diagnostic accuracy, wide availability, and absence of ionizing radiation. However, visualization of the proximal subclavian and brachiocephalic veins may occasionally be limited by the clavicle [37,38]. When clinical suspicion remains high despite negative or inconclusive ultrasonography, cross-sectional imaging with MR venography or CT venography may be considered according to pregnancy status, anatomical site, and local availability.

Therapeutic management does not substantially differ from that of lower-extremity DVT. Low-molecular-weight heparin (LMWH) remains the anticoagulant of choice throughout pregnancy because of its established efficacy and favorable maternal–fetal safety profile [27].

Although uncommon in absolute terms, upper-extremity DVT appears to be disproportionately represented among reported ART/OHSS-associated thrombotic events. Early recognition, prompt imaging, timely initiation of anticoagulation, and multidisciplinary management remain essential to minimize maternal morbidity and prevent potentially life-threatening complications such as pulmonary embolism.

## 8. Pharmacological Treatment in IVF and Splanchnic Vein Thrombosis

Splanchnic vein thrombosis (SVT), including portal vein thrombosis (PVT), mesenteric vein thrombosis (MVT), splenic vein thrombosis, and Budd–Chiari syndrome, represents one of the rarest but potentially most severe thrombotic complications associated with assisted reproductive technologies (ARTs).

The pathogenesis of SVT during ARTs is multifactorial and largely reflects the interaction between ovarian hyperstimulation syndrome (OHSS), pregnancy-associated hypercoagulability, and patient-specific thrombotic predisposition. Severe OHSS induces hemoconcentration, endothelial dysfunction, increased thrombin generation, platelet activation, and reduced venous blood flow, creating a favorable environment for thrombosis in atypical venous districts [4,39]. In women carrying inherited thrombophilia or antiphospholipid syndrome, these mechanisms may synergistically increase the likelihood of thrombus formation within the low-flow splanchnic circulation [4,30].

Unlike lower-extremity deep vein thrombosis, SVT frequently presents with nonspecific symptoms that may delay diagnosis [39]. Persistent or severe abdominal pain, nausea, vomiting, abdominal distension, fever, unexplained ascites, or abnormal liver function tests should raise suspicion, particularly in women with recent ovarian stimulation or severe OHSS. Because clinical findings are often subtle during the initial phase, early imaging plays a pivotal role in preventing intestinal ischemia or progressive portal hypertension [39].

The optimal imaging strategy depends on the vascular territory involved and the clinical presentation. Doppler ultrasonography is useful as an initial non-invasive examination, particularly for portal and hepatic venous thrombosis. However, contrast-enhanced CT is particularly important when mesenteric venous thrombosis or intestinal ischemia is suspected, whereas MR venography may represent an alternative when avoidance of ionizing radiation is desirable.

Low-molecular-weight heparin (LMWH) remains the cornerstone of treatment for acute SVT occurring during pregnancy or following ARTs [40,41].

Because high-quality prospective studies are lacking, current recommendations regarding thromboprophylaxis are largely extrapolated from the general management of pregnancy-associated venous thromboembolism.

Although SVT remains an uncommon complication, increasing awareness among fertility specialists and internists is essential because timely diagnosis and early anticoagulant therapy substantially improve clinical outcomes. Future multicenter registries specifically focusing on atypical thrombosis during ARTs are needed to better define incidence, prognostic factors, and optimal management strategies.

## 9. Pharmacological Treatment in IVF and Cerebral Venous Thrombosis

Cerebral venous thrombosis (CVT) is an uncommon but potentially life-threatening manifestation of venous thromboembolism (VTE), accounting for less than 1% of all cerebrovascular events. Although rare, CVT predominantly affects young women and is strongly associated with conditions characterized by hormonal changes, including pregnancy, the puerperium, oral contraceptive use, and assisted reproductive technologies (ARTs). Among reported cases of CVT associated with IVF, severe OHSS has frequently been described as a concomitant condition; however, available evidence derives predominantly from case reports and small case series and does not permit reliable estimation of its prevalence [42,43].

Unlike lower-extremity deep vein thrombosis, the diagnosis of CVT remains particularly challenging because clinical manifestations are frequently nonspecific. Progressive headache is the most common presenting symptom and may precede neurological deterioration by several days [44]. Focal neurological deficits, seizures, papilledema, altered consciousness, diplopia, and visual disturbances may subsequently develop according to the location and extent of venous occlusion. Because these manifestations overlap with numerous neurological disorders encountered during pregnancy, a high degree of clinical suspicion is required, especially in women with recent ovarian stimulation or severe OHSS [44].

Recent evidence increasingly supports the concept of immunothrombosis, whereby endothelial activation, neutrophil extracellular traps (NETs), platelet–leukocyte interactions, and cytokine release contribute synergistically to thrombus formation [45]. Similar mechanisms have been described in other inflammatory disorders associated with an increased risk of venous thromboembolism, highlighting the close biological interplay between inflammation and coagulation [45].

Magnetic resonance imaging (MRI) combined with MR venography provides high diagnostic accuracy for suspected CVT without ionizing radiation and is particularly attractive during pregnancy. CT with CT venography also has an established diagnostic role and may be preferred according to clinical urgency, availability, and patient-specific circumstances. Laboratory biomarkers such as D-dimer may support clinical suspicion; however, physiological activation of coagulation during pregnancy substantially limits their negative predictive value, and a normal D-dimer concentration cannot safely exclude CVT in pregnant women or those undergoing ARTs [46].

Prompt initiation of therapeutic anticoagulation remains the cornerstone of treatment, even in patients presenting with hemorrhagic venous infarction, as the benefits of preventing thrombus propagation generally outweigh the risk of hematoma expansion. Low-molecular-weight heparin (LMWH) is recommended throughout pregnancy because of its favorable safety profile and inability to cross the placenta [47].

## 10. Pharmacological Treatment in IVF and Arterial Thrombosis

Most reported cases have been described in women who developed severe ovarian hyperstimulation syndrome (OHSS) [48,49], suggesting that profound endothelial dysfunction, hemoconcentration, and systemic activation of coagulation may contribute not only to venous but also to arterial thrombogenesis [50,51,52,53].

Unlike venous thromboembolism, arterial thrombosis generally occurs early during ovarian stimulation or shortly after human chorionic gonadotropin (hCG) administration [40,41,42,43]. Ischemic stroke represents the most frequently reported arterial complication, followed by acute myocardial infarction, retinal artery occlusion, and, more rarely, peripheral arterial thrombosis [40,43,48]. Although the absolute incidence remains extremely low, clinicians should be aware of these events because delayed recognition may result in irreversible neurological or cardiovascular damage [50,54].

The biological mechanisms underlying arterial thrombosis partially differ from those driving venous thromboembolism. In the arterial circulation, endothelial activation or injury promotes exposure of adhesive substrates and release of von Willebrand factor, favoring platelet tethering, adhesion, and activation under high-shear conditions. Activated platelets subsequently release ADP, thromboxane A2, and other mediators that recruit additional platelets and promote formation of a platelet-rich thrombus. OHSS-related hemoconcentration may further increase cellular interactions and blood viscosity, while endothelial dysfunction and vasoactive imbalance may facilitate vasoconstriction or vasospasm. Inflammatory leukocyte–platelet interactions and tissue-factor expression may simultaneously activate thrombin generation, providing an important link between cellular platelet responses and the coagulation cascade. By contrast, venous thrombosis is relatively more dependent on low-flow conditions, coagulation-factor activation, thrombin generation, fibrin deposition, and impaired fibrinolysis. The distinction is therefore not absolute: endothelial dysfunction, inflammation, platelets, leukocytes, and coagulation participate in both processes, but their relative contribution differs according to the vascular territory and local hemodynamic conditions [51,52,53]. Conventional cardiovascular risk factors, including smoking, hypertension, diabetes mellitus, and obesity, may further increase arterial thrombotic risk. APS represents an established acquired thrombophilic condition associated with both venous and arterial thrombosis. In contrast, inherited thrombophilias are predominantly associated with venous thromboembolism, and their role as independent risk factors for arterial thrombosis remains considerably less established [51,54,55,56,57,58,59]. Long-term antithrombotic treatment should be individualized according to the underlying mechanism of the arterial event. Low-dose aspirin is generally considered safe during pregnancy when clinically indicated [50], whereas therapeutic anticoagulation with low-molecular-weight heparin (LMWH) should be reserved for women with an established indication, such as concomitant venous thromboembolism, cardioembolic stroke, or persistent high-risk thrombophilic conditions [60,61]. Current evidence does not support routine antiplatelet therapy in women undergoing ARTs who do not present additional cardiovascular or obstetric indications [60,62].

Overall, arterial thrombosis remains one of the least understood complications of ARTs. Future multicenter registries specifically designed to collect arterial thrombotic events after IVF are needed to clarify incidence, identify predisposing clinical factors, and establish evidence-based preventive and therapeutic strategies [63].

## 11. Different Thrombotic Risk in Women Undergoing Heterologous In Vitro Fertilization Procedures

Data specifically addressing thrombotic risk in heterologous IVF remain scarce, and current evidence is largely extrapolated from studies evaluating ARTs as a whole [54,55,56,57].

Moreover, much of the available evidence in this field specifically concerns pregnancies achieved through oocyte donation rather than heterologous IVF as a homogeneous category; this distinction should be considered when interpreting the available data.

Women undergoing heterologous IVF differ substantially from those receiving homologous IVF. Recipients are generally older and may more frequently present with obesity, chronic hypertension, diabetes mellitus, previous venous thromboembolism, or other cardiovascular comorbidities [64,65,66,67,68]. Advanced maternal age is itself an established risk factor for thromboembolic and cardiovascular complications during pregnancy and may partly account for the vascular events observed in this population [64,68,69,70].

Furthermore, preparation of the endometrium for embryo transfer frequently requires administration of exogenous estrogens and progesterone. In programmed cycles without a corpus luteum, hormonal exposure and altered vascular adaptation may contribute to endothelial and hemodynamic changes, although a direct relationship with maternal thrombosis has not been conclusively demonstrated [69,71,72].

A summary of the suggested thromboprophylactic strategies according to different clinical scenarios in women undergoing assisted reproductive technologies, including ovarian hyperstimulation syndrome, previous venous thromboembolism, inherited thrombophilia, antiphospholipid syndrome, and heterologous IVF, is provided in Table 3.

Pregnancies achieved through oocyte donation are consistently associated with an increased frequency of hypertensive disorders, particularly gestational hypertension, pre-eclampsia, and severe pre-eclampsia [64,65,66,67,68,69,70]. These findings suggest that heterologous pregnancies may have a distinctive cardiovascular, endothelial, placental, and immunological risk profile. However, the increased incidence of hypertensive disorders should not be interpreted as direct evidence that oocyte donation independently causes venous or arterial thrombosis.

It therefore remains unclear whether heterologous IVF intrinsically increases thrombotic risk or whether any apparent increase primarily reflects differences in recipient age, cardiovascular comorbidities, hormonal regimens, and pregnancy-related complications. Most available studies are retrospective, include heterogeneous populations, and were primarily designed to evaluate obstetric rather than thrombotic outcomes [64,65,66,67,68,69,70]. Consequently, there is currently insufficient evidence to consider oocyte-donation IVF, in isolation, an indication for pharmacological thromboprophylaxis.

Another important aspect concerns multidisciplinary evaluation. Women undergoing heterologous IVF may present with more complex medical histories than younger women receiving homologous IVF, making collaboration among reproductive medicine specialists, hematologists, internists, maternal–fetal medicine physicians, and cardiologists particularly valuable [71,72]. Such an approach permits individualized assessment of thrombotic, cardiovascular, and obstetric risks while minimizing unnecessary anticoagulant exposure.

Overall, the independent contribution of heterologous IVF or oocyte donation to thrombotic risk remains uncertain. Maternal characteristics, hormonal exposure, cardiovascular comorbidities, and pregnancy-related complications are likely to contribute, but current evidence does not allow their effects to be reliably separated from those of the reproductive procedure itself. Well-designed prospective studies are needed to clarify these relationships.

## 12. Discussion

Thrombotic complications associated with assisted reproductive technologies represent a unique clinical scenario in which physiological, iatrogenic, and patient-specific factors converge to generate a transient but potentially severe hypercoagulable state [51,52,53,54,55,56,57]. Although the absolute incidence of venous thromboembolism remains relatively low, the continuous expansion of IVF worldwide implies that clinicians will increasingly encounter these complications in clinical practice. Available evidence consistently identifies OHSS as the principal treatment-related determinant of thrombosis, whereas inherited thrombophilia, antiphospholipid syndrome, obesity, advanced maternal age, cardiovascular comorbidities, and previous VTE substantially modify individual risk [51,52,53,54,55,56,57].

Importantly, contemporary reproductive strategies are modifying this risk landscape. Wider adoption of individualized ovarian stimulation, GnRH-antagonist protocols, GnRH-agonist triggering, freeze-all strategies, and, in selected high-risk women, approaches minimizing gonadotropin exposure have substantially changed the prevention of severe OHSS [8]. Furthermore, contemporary epidemiological evidence indicates that thrombotic risk is not uniform across ART strategies, with fresh embryo-transfer pregnancies showing a substantially greater first-trimester VTE risk than frozen-thawed embryo-transfer pregnancies [31].

An observation emerging from the available literature is the relatively frequent reporting of thrombotic events at unusual anatomical sites in association with ARTs and OHSS. Reported ART-associated thrombotic events have involved the internal jugular, subclavian, cerebral, portal, mesenteric, and hepatic veins [51,52,53,54,55,56,57,58,59,60,61,62,63]. However, these observations derive largely from case reports and small series and should not be interpreted as establishing a distinct anatomical pattern of ART-associated thrombosis. Although these observations are clinically relevant, available evidence is insufficient to define ART-associated thrombosis as a separate thrombotic phenotype. Rather, unusual-site thromboses appear to be relatively overrepresented among reported ART/OHSS-associated events and should therefore remain within the clinician’s diagnostic awareness.

Another important aspect concerns thromboprophylaxis. Available recommendations do not support routine anticoagulation for all women undergoing IVF and instead favor individualized risk assessment [51,61,72]. This approach appears justified because most women undergoing ARTs will never experience thrombotic complications, whereas unnecessary anticoagulation may increase bleeding, bruising, treatment burden, and obstetric complications. At present, no validated ART-specific model or biomarker strategy is available to identify women who will benefit from prophylactic LMWH. Development and prospective validation of such tools should therefore be considered a future research priority.

Thrombo-inflammation may contribute to ART-associated thrombosis through interactions among endothelial dysfunction, platelet activation, inflammatory mediators, and coagulation. Mechanisms such as neutrophil extracellular traps, platelet–leukocyte interactions, and complement activation have been implicated in thrombotic disease more broadly; however, their specific contribution to ART/OHSS-associated thrombosis remains incompletely characterized and is largely extrapolated from other thrombo-inflammatory conditions [73,74,75,76,77,78,79].

Despite substantial progress in reproductive medicine, important knowledge gaps remain. Most available evidence derives from retrospective cohorts, observational databases, case series, reviews, and isolated case reports. Randomized clinical trials evaluating thromboprophylaxis specifically in women undergoing IVF remain extremely limited. Furthermore, available studies frequently combine heterogeneous populations, ovarian-stimulation protocols, hormonal regimens, and definitions of OHSS, making direct comparisons difficult. The absence of validated risk-prediction models specifically developed for women undergoing ARTs continues to represent one of the principal limitations of current clinical practice.

The present review also has methodological limitations inherent to its narrative design. Although a structured literature search was performed, study selection was not conducted according to a systematic-review protocol, and no formal risk-of-bias assessment was undertaken. Furthermore, the certainty of the available evidence was not formally assessed using the Grading of Recommendations Assessment, Development and Evaluation (GRADE) framework. Therefore, the clinical statements discussed in this review should not be interpreted as formally graded recommendations. This limitation is particularly relevant because ART-specific evidence is heterogeneous and several management strategies are necessarily extrapolated from pregnancy-associated or general VTE populations.

Future research should prioritize prospective multicenter registries and the development and external validation of ART-specific thrombotic risk-prediction models. The potential contribution of biomarkers and other individualized risk markers remains exploratory and requires prospective validation before incorporation into clinical decision-making.

## 13. Conclusions

Assisted reproductive technologies have profoundly transformed the management of infertility, allowing millions of women worldwide to achieve pregnancy. Although thrombotic complications remain relatively uncommon, they continue to represent one of the most clinically significant adverse events associated with ovarian stimulation and early pregnancy because of their potential impact on maternal morbidity and mortality. The available evidence indicates that thrombotic risk is not uniformly distributed among women undergoing IVF but rather results from the complex interaction between treatment-related factors, pregnancy-associated physiological hypercoagulability, inherited or acquired thrombophilia, and individual patient characteristics.

Among treatment-related complications, OHSS remains the principal determinant of thrombotic events because of the profound endothelial dysfunction, hemoconcentration, and coagulation activation that characterize this condition. In addition to conventional lower-extremity DVT and PE, unusual-site venous thromboses involving the upper-extremity, cerebral, and splanchnic circulation appear to be relatively overrepresented among reported ART/OHSS-associated events.

Routine pharmacological thromboprophylaxis is not indicated for all women undergoing ARTs. Prevention should instead be based on individualized assessment of previous VTE, APS, inherited thrombophilia, OHSS, pregnancy status, and additional thrombotic risk factors. Importantly, ART-specific evidence remains limited, and several current management recommendations are necessarily extrapolated from pregnancy-associated or general VTE guidelines. Prospective multicenter studies and validated ART-specific risk-assessment models are needed to strengthen the evidence base and better identify women who may benefit from thromboprophylaxis.

## Figures and Tables

**Table 1:** Major Risk Factors for Venous Thromboembolism During Assisted Reproductive Technologies.

Risk Factor	Mechanism	Clinical Implications
Previous VTE	Persistent prothrombotic tendency	Strong indication for individualized thromboprophylaxis
Severe ovarian hyperstimulation syndrome (OHSS)	Hemoconcentration, endothelial dysfunction, VEGF-mediated vascular permeability, coagulation activation	Major treatment-related risk factor; prophylactic LMWH generally recommended
Antiphospholipid syndrome (APS)	Autoimmune activation of coagulation and endothelial dysfunction	Requires individualized anticoagulation strategy
High-risk inherited thrombophilia (e.g., antithrombin deficiency, homozygous Factor V Leiden or Prothrombin G20210A mutation)	Increased thrombin generation	Consider prophylactic LMWH during ARTs and pregnancy
Combined inherited/acquired thrombophilia	Synergistic procoagulant effects	Comprehensive hematological evaluation recommended
Pregnancy after IVF	Physiological hypercoagulability and venous stasis	Risk highest during the first trimester
Advanced maternal age	Endothelial dysfunction and increased cardiovascular risk	Risk assessment should include age-related comorbidities
Obesity	Chronic inflammation, impaired fibrinolysis, venous stasis	Weight optimization before ARTs is advisable
Prolonged immobilization	Venous stasis	Mechanical and/or pharmacological prophylaxis when appropriate
Smoking	Endothelial injury and platelet activation	Smoking cessation should be encouraged before ART
Multiple pregnancy	Increased physiological hypercoagulability	Closer clinical surveillance
Cesarean delivery	Surgical trauma and postpartum hypercoagulability	Postpartum thromboprophylaxis according to guidelines
Active malignancy	Cancer-associated thrombosis	Individualized anticoagulant management
Chronic inflammatory diseases	Persistent endothelial activation and thrombo-inflammation	Evaluate overall thrombotic profile

Legend. The table summarizes the principal patient-related and treatment-related risk factors associated with venous thromboembolism (VTE) during assisted reproductive technologies (ARTs). Ovarian hyperstimulation syndrome (OHSS), previous VTE, antiphospholipid syndrome, and high-risk inherited thrombophilia represent the strongest determinants of thrombotic risk and should prompt individualized assessment before ovarian stimulation. Abbreviations: ARTs, assisted reproductive technologies; APS, antiphospholipid syndrome; IVF, in vitro fertilization; LMWH, low-molecular-weight heparin; OHSS, ovarian hyperstimulation syndrome; VEGF, vascular endothelial growth factor; VTE, venous thromboembolism.

**Table 2:** Clinical Characteristics, Diagnosis, and Management of Thrombotic Complications Associated with Assisted Reproductive Technologies.

Thrombotic Manifestation	Typical Clinical Presentation	Preferred Diagnostic Modality	First-Line Treatment	Prognosis/Clinical Considerations
Lower-extremity deep vein thrombosis (DVT)	Unilateral leg swelling, pain, erythema, tenderness	Compression duplex ultrasonography	Therapeutic LMWH	Most frequent thrombotic complication; favorable prognosis with early treatment
Pulmonary embolism (PE)	Dyspnea, pleuritic chest pain, tachycardia, hypoxemia, syncope	CTPA or ventilation–perfusion (V/Q) scan according to pregnancy-specific diagnostic algorithms	Therapeutic LMWH; thrombolysis only in high-risk PE	Major cause of maternal morbidity and mortality
Upper-extremity DVT	Arm swelling, neck pain, supraclavicular edema, collateral veins	Duplex ultrasonography ± CT/MR venography	Therapeutic LMWH	More common after ARTs than in spontaneous pregnancy; often associated with OHSS
Cerebral venous thrombosis (CVT)	Persistent headache, seizures, focal neurological deficits, papilledema	MRI with MR venography	Therapeutic LMWH even in the presence of hemorrhagic venous infarction	Requires prompt diagnosis; neurological sequelae may occur
Splanchnic vein thrombosis (portal, mesenteric, splenic veins, Budd–Chiari syndrome)	Abdominal pain, ascites, nausea, abnormal liver function tests	Doppler ultrasonography, contrast-enhanced CT or MR venography	Therapeutic LMWH; surgery or endovascular treatment in selected cases	Rare but potentially life-threatening if diagnosis is delayed
Arterial thrombosis (ischemic stroke, myocardial infarction, peripheral arterial thrombosis)	Acute neurological deficit, chest pain, limb ischemia	CT/MRI brain, coronary angiography, vascular imaging according to site	Standard reperfusion therapy plus pregnancy-adapted antithrombotic management	Extremely uncommon but associated with high morbidity

Legend. The table summarizes the principal thrombotic manifestations reported during assisted reproductive technologies (ARTs), including their typical clinical presentation, preferred diagnostic approach, first-line therapeutic strategy, and major prognostic considerations. Venous thromboembolism predominates, while thrombosis involving unusual venous sites—including the upper-extremity, cerebral, and splanchnic circulation—appears to be relatively overrepresented among reported ART/OHSS-associated thrombotic events. Abbreviations: ARTs, assisted reproductive technologies; CTPA, computed tomography pulmonary angiography; CT, computed tomography; CVT, cerebral venous thrombosis; DVT, deep vein thrombosis; LMWH, low-molecular-weight heparin; MR, magnetic resonance; MRI, magnetic resonance imaging; OHSS, ovarian hyperstimulation syndrome; PE, pulmonary embolism; V/Q, ventilation–perfusion.

**Table 3:** Suggested Management According to Clinical Scenario in Women Undergoing Assisted Reproductive Technologies.

Clinical Scenario	Suggested Thromboprophylaxis	Comments	Evidence Basis/Supporting References
No thrombotic risk factors undergoing ART	No pharmacological prophylaxis	Early mobilization and adequate hydration are recommended.	ART-specific evidence/individualized approach [17,51,61,72]
Mild OHSS	Usually no LMWH	Individual assessment according to additional risk factors.	ART/OHSS-specific evidence; individualized approach [15,51,61]
Severe OHSS	Prophylactic LMWH	Continue until resolution of OHSS; extension during pregnancy should be individualized.	ART/OHSS-specific evidence and guideline-based recommendations [15,51,61]
Previous VTE (provoked by a transient major risk factor)	Consider prophylactic LMWH	Decision should consider recurrence risk and additional pregnancy-related factors.	Extrapolated from pregnancy-associated VTE guidelines [25]
Previous unprovoked VTE or hormone-related VTE	Prophylactic LMWH	Continue throughout pregnancy and postpartum according to current guidelines.	Extrapolated from pregnancy-associated VTE guidelines [25]
Previous recurrent VTE	Prophylactic LMWH; dose intensity should be individualized	Routine intermediate-dose LMWH has not demonstrated clear superiority over low-dose prophylaxis; multidisciplinary assessment is recommended	Pregnancy-associated VTE evidence/guidelines [25,26]
High-risk inherited thrombophilia (e.g., antithrombin deficiency, homozygous Factor V Leiden or Prothrombin G20210A mutation)	Prophylactic LMWH	Particularly if additional risk factors are present.	Extrapolated from pregnancy-associated VTE guidelines [17,25]
Low-risk inherited thrombophilia without previous VTE	Usually no routine LMWH	Clinical surveillance; prophylaxis only if additional risk factors coexist.	Extrapolated from pregnancy-associated VTE guidelines [17,25]
Obstetric APS without previous thrombosis	Low-dose aspirin plus prophylactic-dose LMWH	Management should follow obstetric APS recommendations and be individualized according to the obstetric history and antiphospholipid antibody profile.	APS/pregnancy-specific guidelines [6]
Thrombotic APS (previous arterial or venous thrombosis)	Therapeutic-dose LMWH during pregnancy, with low-dose aspirin when indicated	Anticoagulant intensity should reflect the previous thrombotic phenotype and current APS-specific recommendations; specialist management is advised.	APS/pregnancy-specific guidelines [6]
Obesity (BMI ≥ 30 kg/m^2^) with additional risk factors	Consider prophylactic LMWH	Weight optimization before ARTs is encouraged.	Extrapolated from pregnancy-associated VTE guidelines [25]
Prolonged immobilization or hospitalization	Prophylactic LMWH	Resume early mobilization whenever possible.	Extrapolated from pregnancy-associated/general VTE guidelines [25]
Heterologous IVF without additional thrombotic risk factors	No routine pharmacological prophylaxis	Risk assessment should be based on maternal characteristics rather than IVF technique alone.	ART-specific observational evidence/individualized assessment [71,72]
Postpartum after ARTs in women at increased thrombotic risk	Continue LMWH for at least 6 weeks	Postpartum represents the period of highest thrombotic risk.	Extrapolated from pregnancy-associated VTE guidelines [25]

Legend. This table summarizes a practical approach to thromboprophylaxis in women undergoing assisted reproductive technologies (ARTs). No validated ART-specific thrombotic risk-stratification model is currently available. Management should therefore be individualized according to previous VTE, the circumstances of previous thrombotic events, thrombophilia, antiphospholipid syndrome, ovarian hyperstimulation syndrome, pregnancy status, and concomitant risk factors. Some recommendations are derived from ART/OHSS-specific evidence, whereas others are extrapolated from guidelines addressing pregnancy-associated VTE. Routine pharmacological thromboprophylaxis is not recommended for all women undergoing ARTs. The evidence basis for each recommendation is indicated in the table. ART/OHSS-specific recommendations are distinguished from recommendations extrapolated from pregnancy-associated or general VTE guidelines, reflecting the limited availability of direct evidence in women undergoing ARTs. Abbreviations: APS, antiphospholipid syndrome; ARTs, assisted reproductive technologies; body mass index (BMI), body mass index; IVF, in vitro fertilization; LMWH, low-molecular-weight heparin; OHSS, ovarian hyperstimulation syndrome; VTE, venous thromboembolism.

## Data Availability

No new data were created or analyzed in this study. Data sharing is not applicable to this article.

## Abbreviations

APS, antiphospholipid syndrome; ARTs, assisted reproductive technologies; BMI, body mass index; CT, computed tomography; CTPA, computed tomography pulmonary angiography; CVT, cerebral venous thrombosis; DVT, deep vein thrombosis; GnRH, gonadotropin-releasing hormone; hCG, human chorionic gonadotropin; IVF, in vitro fertilization; IVM, in vitro maturation; LMWH, low-molecular-weight heparin; MR, magnetic resonance; MRI, magnetic resonance imaging; MVT, mesenteric vein thrombosis; NETs, neutrophil extracellular traps; OHSS, ovarian hyperstimulation syndrome; PCOS, polycystic ovary syndrome; PE, pulmonary embolism; PVT, portal vein thrombosis; SVT, splanchnic vein thrombosis; VEGF, vascular endothelial growth factor; V/Q, ventilation–perfusion; VTE, venous thromboembolism.
